# SWEET: A Realistic Multiwavelength 3D Simulator for Automotive Perceptive Sensors in Foggy Conditions

**DOI:** 10.3390/jimaging9020054

**Published:** 2023-02-20

**Authors:** Amine Ben-Daoued, Pierre Duthon, Frédéric Bernardin

**Affiliations:** Cerema, Research Team “Intelligent Transport Systems”, 8-10 Rue Bernard Palissy, CEDEX 2, F-63017 Clermont-Ferrand, France

**Keywords:** fog, Koschmieder, Monte Carlo simulation

## Abstract

Improving the reliability of automotive perceptive sensors in degraded weather conditions, including fog, is an important issue for road safety and the development of automated driving. Cerema has designed the PAVIN platform reproducing fog and rain conditions to evaluate optical automotive sensor performance under these conditions. In order to increase the variety of scenarios and technologies under test, the use of digital simulation becomes a major asset. The purpose of this paper is to revive the debate around the realism of the various models underlying the numerical methods. The simulation of the radiative transfer equation by Monte Carlo methods and by simplified noise models is examined. The results of this paper show some gaps in foggy scenes between the ray-tracing method, which is considered to be the most realistic, and simple models for contrast evaluation, which can have a particularly strong impact on obstacle detection algorithms.

## 1. Introduction

The development of automated mobility is at the heart of many roadmaps of professional associations in the mobility sector or of the institutions responsible for public economic and societal development policies [1,2]. These roadmaps highlight in particular the driving scenario approach to demonstrating the safety of automated road transport systems, even including scenarios based on digital simulation, taking into account degraded weather conditions among the various layers (infrastructure, objects on the road, manoeuvres, etc.) used to describe a scenario (French ADScene-MOSAR, German PEGASUS, Dutch StreetWise, Israeli CDV projects, etc.).

The development of automated vehicles requires the integration of several driving assistance technologies based on optical devices such as radar, LiDAR, camera, etc. These are used for obstacle detection, pedestrian detection, traffic sign recognition, trajectory correction, etc. The safety related to the performance of these devices is a major issue. It is well-known that these devices are perturbed by adverse weather conditions such as rain, snow, or fog [3,4,5,6,7,8]. Consequently, automotive suppliers and car manufacturers are overall interested in making the various technologies more reliable by testing them under extreme conditions and in the most exhaustive possible situations [4]. To achieve this, perception and driving assistance sensors, operating at different wavelengths, require the various meteorological conditions to be considered in addition to a large variety of targets to detect or recognize in the road scene. Then, testing these advanced and complex technologies is generally not feasible based only on experimental work. For this reason, digital simulation solutions are developed to massively test new and varied technologies under the most exhaustive conditions, which is not possible exclusively experimentally.

The Horizon Europe ROADVIEW project (Robust Automated Driving in Extreme Weather), supporting this work, aims to address the impact of harsh weather conditions on automotive perception sensors. Work Package 3, devoted to digital simulation, will develop sensor noise modelling to represent the variations in harsh weather characteristics and thus generate realistic synthetic sensor data. The goal is to build automated vehicle testing by simulation-assisted methods.

Cerema, equipped with its fog and rain PAVIN platform [9,10], has carried out work for years with several national and international partners aimed at better understanding the behaviour of the perception systems of intelligent vehicles under harsh weather conditions [11,12]. Additionally, in support of its experimental work, Cerema is also interested in the theoretical aspects of electromagnetic wave propagation under adverse meteorological conditions, including fog, in order to meet the challenges of numerical simulation of autonomous vehicles in all weather conditions [13].

Nowadays, there exist optical sensor simulators (for camera, radar and LiDAR) that consider atmospheric conditions such as fog or rain. We can cite some software: Ansys AVxcelerate Sensors [14], Carla Simulator [15,16], Pro-SiVIC [17,18,19], Electro-Optics sensor simulation [20,21], AVSimulation [22], 4DVirtualiz [23], Mitsuba [24], etc. Different electromagnetic wave propagation models are used in these simulators, ranging from simple Beer–Lambert type attenuation laws to complex offline ray-tracing simulations.

Numerical simulation plays an important role in the field of automated vehicles. Indeed, it reduces the costs of massive sensor testing, while ensuring optimal safety. This also makes certain dangerous scenarios feasible, such as testing a pedestrian detection system requiring the presence of a pedestrian in real-world simulation, thus risking being run over during the experiment. In several applications and in particular in those related to automated vehicles, the use of real-time simulation is an important issue because it provides access to all the simulation variables in real time. This instantaneous access is necessary in some cases such as X-in-the-loop applications to test sensors [25]. Most real-time simulators in the automotive and computer science community use Koschmieder’s model (from the Beer–Lambert method) to foggify sensor data [26,27,28,29], but only a few studies have addressed the question of the realism of the Koschmieder model among other fog models [28,29].

The main goal of this work is to revive the debate about the realism of fog models by comparing a simple commonly used 1D simulator based on Beer–Lambert type attenuation laws and a full Monte Carlo simulator developed by Cerema, called SWEET (Simulating WEather for intElligent Transportation systems). More specifically, we are interested in comparing the realism of simulated camera images in the visible wavelength range for two scenes (intra-urban scene and extra-urban scene) based on the SWEET simulator and on the simplified 1D model. In order to also compare our work to the literature, we also chose to compare our SWEET simulator to the Mitsuba simulator. Finally, there are studies in the literature that have addressed the impact of degraded meteorological conditions on other types of cameras operating in other wavelength ranges (NIR, SWIR, LWIR) [30]. These wavelengths are not the subject of this paper, but we will certainly address them in future work.

Section 2 details the mathematical background of the simulation tools which are compared: ray-tracing methods for the 3D radiative transfer simulation and Koschmieder law-based harsh weather noises. Section 3 describes the applications of the simulation methods to foggy scenes by camera imaging. Section 4 and Section 5 show the results of the investigation, respectively, by a visual comparison between the different simulation methods and by a more quantitative comparison based on relevant metrics for contrast assessment. Finally, Section 6 offers a conclusion to the study.

## 2. Mathematical Background for Modelling and Numerical Simulation

In order to model perception sensors, it is necessary to be able to model the path of a light ray in space, including under harsh weather conditions, such as fog. The propagation of electromagnetic waves in participating media, such as fog, is governed by the radiative transfer equation (RTE) in which the optical parameters (related to scattering, absorption and extinction) of the medium are considered. Assuming unpolarized light, by using the Mie scattering theory [31], these wavelength-dependent parameters can be computed thanks to the droplet size distribution (DSD) of the fog.

The RTE serves to simulate the radiance $L_{\lambda }\left(t,r,u\right)$ corresponding, for a wavelength λ, to the intensity of the electromagnetic energy flux (in W) of the radiation propagating in the direction *u*, per unit of area (in m${}^{2}$) perpendicular to the direction of propagation, per unit of solid angle (in sr) and per unit of wavelength (in microns), and expressed in $W/m^{2}/\mu /sr$.

We recall here the RTE [31]:

(1) $$\begin{array}{c} \frac{1}{c}\frac{\partial L_{\lambda }}{\partial t}\left(t,r,u\right)+u\cdot \nabla_{r}L_{\lambda }\left(t,r,u\right)=\\ -\sigma_{\lambda }\left(r\right)L_{\lambda }\left(t,r,u\right)-\kappa_{\lambda }\left(r\right)L_{\lambda }\left(t,r,u\right)+\frac{\sigma_{\lambda }\left(r\right)}{4\pi }\int_{S^{2}}L_{\lambda }\left(t,r,v\right)\Phi_{\lambda }\left(r,v,u\right)dv+q(t,r,u), \end{array}$$

where *c* is the speed of light, *t*, *r*, *u*, $\sigma_{\lambda }$, $\kappa_{\lambda }$, $\Phi_{\lambda }$ and q(t,x,u) denote, respectively, the time, the position in space, the wave propagation direction, the scattering coefficient, the absorption coefficient, the phase function for the wavelength λ and a space continuous source. The three-dimensional unit sphere is denoted by $S^{2}$. By using the vocabulary dedicated to the rendering volumetric path tracing method, the terms in blue and green represent the out-scattering and absorption terms, respectively. These two terms together form the extinction term. The term in red corresponds to the in-scattering term, which is the most expensive part to calculate as it requires integration over all paths in the scene. Finally, the term in brown is for emission.

Optical passive objects and local light sources are taken into account thanks to the boundary conditions of Equation (1). For each light source occupying the space region *S* and emitting light from its surface ∂S, we have:

(2) $$\forall \;r\in \partial S,\;\forall \;u\in S^{2},\;u\cdot n_{r}^{S}>0,\;L_{\lambda }\left(t,r,u\right)=E_{\lambda }^{S}\left(t,r,u\right),$$

where $n_{r}^{S}$ denotes the outward normal vector of *S* at point r∈∂S, and $E_{\lambda }$ is given. For each passive object occupying the space region *O* with a surface denoted by ∂O, we have:

(3) $$\forall \;r\in \partial O,\;\forall \;u\in S^{2},\;u\cdot n_{r}^{O}>0,\;L_{\lambda }\left(t,r,u\right)=\int_{v\in S^{2},\;v\cdot n_{r}^{O}<0}L_{\lambda }\left(t,r,v\right)B_{\lambda }^{O}\left(r,v,u\right)dv,$$

where $n_{r}^{O}$ denotes the outward normal vector of *O* at point *r*, and $B_{\lambda }^{O}\left(r,\cdot ,\cdot \right)$ is the BRDF of object O at point r∈∂O.

In the following, we will focus on the stationary case of Equation (1) with a homogeneous fog ($\sigma_{\lambda }$, $\kappa_{\lambda }$ and $\Phi_{\lambda }$ do not depend on r) and a phase function depending only on v·u (scalar product), hence:

(4) $$u\cdot \nabla_{r}L_{\lambda }\left(r,u\right)=-\beta_{\lambda }L_{\lambda }\left(r,u\right)+\frac{\sigma_{\lambda }}{4\pi }\int_{S^{2}}L_{\lambda }\left(r,v\right)\Phi_{\lambda }\left(v\cdot u\right)dv+q\left(r,u\right),$$

where we note $\beta_{\lambda }=\sigma_{\lambda }+\kappa_{\lambda }$ the extinction coefficient at the wavelength λ. Boundary conditions related to this stationary case are given by Equations (2) and (3) in which the time *t* is removed. The physical significance of the phase function $\Phi_{\lambda }$ is important. Indeed, for a photon moving at the speed of light in a medium, the phase function gives the probability of the resulting direction of this particle when it interacts with a water droplet. From an energy point of view, coefficients $\sigma_{\lambda }$ and $\kappa_{\lambda }$ define the amount of energy scattered and absorbed by the water droplets. These optical characteristics of the medium ($\sigma_{\lambda }$, $\kappa_{\lambda }$ and $\Phi_{\lambda }$) are determined according to Mie theory and DSD measurements, under the assumption of spherical water droplets.

**Figure 1:**
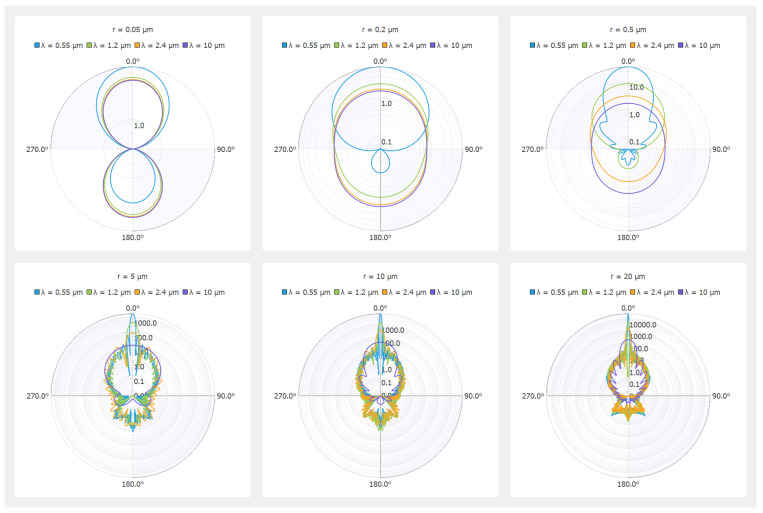
Polar representation of the phase function for six radii (r) of spherical particles and different wavelengths (λ).

The phase function $\Phi_{\lambda }$ for six radii of spherical water droplets and different wavelengths from the visible to the thermal infrared range is shown in polar coordinates in Figure 1. One angle θ=u·v is sufficient to represent this phase function due to the spherical symmetry. We can notice a very weak influence of the wavelength on the phase function for small spheres (r = 0.05 μm and r = 0.2 μm), which is in accordance with Rayleigh’s theory under unpolarized incident light [31,32]. On the other hand, for sphere radii beyond 0.5 μm, the influence of the wavelength is noticeable, and backscattering gradually disappears. Finally, it should be noted that all of the curves presented in Figure 1 consider the variation in the complex refractive index of water according to the wavelength. This shows the importance of using accurate microscopic fog data (actually the measured DSD), not just approximate models simply recalibrated on macroscopic fog density, as already explained [13].

The SWEET simulator developed by Cerema is a research-oriented and physically based simulator for internal use complementary to the Fog and Rain PAVIN platform. It is written in C++, runs on Linux and Microsoft Windows and uses OpenCL for GPU computing. Optimisation techniques such as a SAH kd-Tree acceleration technique of order O(N log N) (N being the number of triangular faces of a scene) are used. More details on these kd-Trees can be found in [33]. For the moment, the whole simulator is fully developed in-house at Cerema. Future work will allow the use of heavily optimized resources, such as the Embree library [34]. We wanted to develop this homemade tool, designed for applications related to the perceptual sensors of autonomous vehicles under adverse weather conditions, in order to be able to add building blocks and to be less dependent on other simulators.

SWEET parallelisation is performed on the rays and not on the volumes of 3D space. Indeed, the rays are distributed on the GPU calculation units. The simulator is designed to be as conservative as possible regarding memory consumption. The rate of convergence of the Monte Carlo algorithm is of order O$\left(1/\sqrt{N}\right)$, N being the number of traced rays. The computation time varies according to the complexity of the scene and the density of the participating medium: highly reflective materials and dense fog conditions slow down the convergence since in these cases the photons are likely to undergo multi-collision and multi-reflection. As an example, on a computer with an NVIDIA GeForce GTX 1080 graphic board, 12 CPU cores and 64 GB of memory, it takes 2 s to path-trace 10 million rays in a relatively complex scene (1.27 Million triangles) with a sparse isotropic participating medium. For a dense isotropic medium in the same scene, the same calculation takes 3 s.

The current version of SWEET only supports Lambertian surfaces; textures are not taken into account for the moment. Ongoing work will enable BSDFs for different types of scattering surfaces. The scattering of the participating medium is supported through measured/computed phase functions (tabbed functions). More scattering models will be implemented in the future (isotropic, Henyey–Greenstein, micro-flakes, etc.). SWEET supports the COLLADA file format, and additional integration is planned in the future. As the characteristics of the participating medium and those of the materials can depend on the wavelength, SWEET is spectral, and its calculations can be made in the range of visible wavelengths (VIS) as well as all wavelengths ranging from VIS up to thermal infrared (LWIR).

SWEET solves the RTE by using a (backward) Monte Carlo method based on full volumetric path tracing in a participating medium: a probabilistic representation of Equation (4) is provided by introducing a stochastic process mimicking photons moving in the medium and being able to interact with droplets of fog [31,35]. More precisely, a ray is sampled from the observation location before being traced along a random direction at the velocity of light *c* during a random duration τ following an exponential probabilistic law (the probability that τ is greater than a given deterministic value *t* is equal to $e^{-\sigma_{\lambda }t}$). If the photon reaches a light source, the random walk is finished. If it reaches an object, the reflective surface properties of this object are taken into account to modify the direction of the photon. If no source or object is reached, a new direction is sampled thanks to the phase function $\Phi_{\lambda }$ (collision with a water droplet) and a new duration $\tau^{\prime }$. The algorithm will continue until a source or an absorbing material is reached.

**Figure 2:**
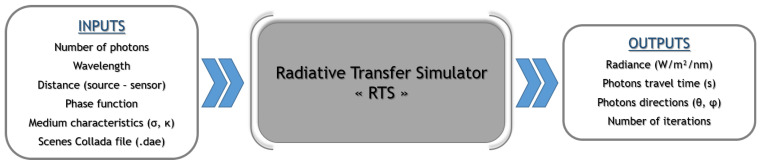
Illustration of the main inputs–outputs of the SWEET simulator.

The main inputs/outputs of SWEET are illustrated in Figure 2.

**Figure 3:**
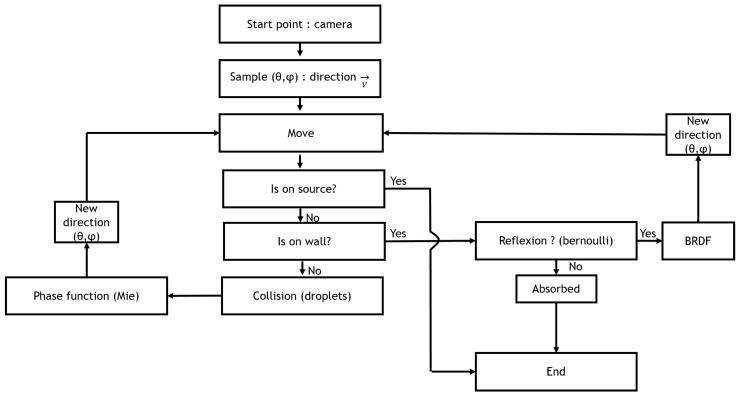
Diagram of the radiative transfer equation simulation method.

The implementation of the SWEET Monte Carlo computing engine generates N independent realisations according to the flowchart in Figure 3. In this flowchart, we see that the photon advances step-by-step in 3D space. At each step, we check whether it encounters a droplet of water or an obstacle in its path. If this is the case, we then evaluate whether the photon is totally absorbed or if it is deviated, then move on to the next iteration. For each realisation (simulation), we can calculate a weighted radiance. The average of all weighted radiance values gives an approximation of the radiance $L_{\lambda }\left(r,\theta ,\phi \right)$. Millions of paths need to be traced to obtain a good quality result without much noise.

A particular case of Equation (4) is often used in a way to achieve an analytical solution. It consists of eliminating the collision term and assuming a constant source *q*. In this case, assuming there is no object and no local source between points $r_{0}$ and $r=r_{0}+xu$ for *x* a real and $u\in S^{2}$, we have:

(5) $$L_{\lambda }\left(r_{0}+xu,u\right)=L_{\lambda }\left(r_{0},u\right)e^{-\beta_{\lambda }x}+\frac{q}{\beta_{\lambda }}\left(1-e^{-\beta_{\lambda }x}\right),$$

leading to the Beer–Lambert solution if q=0:

(6) $$L_{\lambda }\left(r_{0}+xu,u\right)=L_{\lambda }\left(r_{0},u\right)e^{-\beta_{\lambda }x}.$$

The simple case presented above corresponds to the framework of the Koschmieder theory [36,37] allowing the contrast between a black object and a sky background to be evaluated based on visibility attenuation due to the extinction of the medium between the object and the observer. This theory is used in image processing to artificially add fog to an image: the intensity I(x,y) of a pixel (x,y) is linked to the intensity $I_{0}\left(x,y\right)$ without fog and an air–light intensity $I_{s}$:

(7) $$I\left(x,y\right)=I_{0}\left(x,y\right)\;e^{-\beta d(x,y)}+I_{s}\left(1-e^{-\beta d(x,y)}\right),$$

where d(x,y) is the real-world distance between the observer (camera) and the real point associated with the pixel (x,y), and β is the extinction coefficient of the fog for the visible range (λ≃ 550 nm). In the sequel, we will call the hazing image method Equation (7) the 1D model or indifferently the Koschmieder model.

The visibility, or meteorological optical range (MOR), is the distance for which the luminous flux of a collimated light beam is reduced to 5% of its original value [38,39]. According to this definition, the visibility MOR is related to the extinction coefficient β as follows:

(8) $$MOR=\frac{-ln(0.05)}{\beta }\approx \frac{3}{\beta },$$

It is important to mention that in Equation (8) β is considered in the visible band and is assumed constant by the WMO [38]. This is not relevant for infrared wavelengths [40].

We can note that the 1D model Equation (7) can simulate a foggy scene if a previous image without fog is available, which is not the case for the Monte-Carlo-based 3D method.

The SWEET simulator and the 1D model are therefore implemented and used to model different use cases that we define in the following section.

## 3. Use Cases

**Figure 4:**
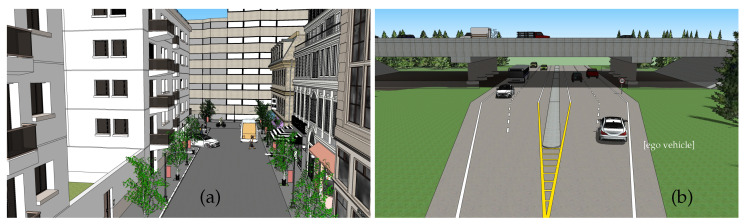
Collada-type models for the road scenes (intra-urban (**a**) and extra-urban (**b**)).

The SWEET simulator can be used in different ways depending on what the user wants to compute, whether it concerns physical quantities such as radiance and irradiance, in any wavelength domain, or the simulation of an entire sensor (i.e., a camera, a LiDAR, etc.). Here we present two use cases for the simulation of a 3D road scene with a camera (operating in the visible light range), as shown in Figure 4. The camera (pinhole model) is placed at the front of the ego vehicle for the extra-urban scene (Figure 4b) which is moving as in real conditions, and at the street entrance for the intra-urban scene (Figure 4a). Both day/night and different fog conditions are experimented.

We first apply the SWEET simulator without fog in order to build a reference image, which is needed by the 1D model (Koschmieder) to obtain a foggy image. On the other hand, the scene is simulated again by using SWEET with foggy conditions. The results are compared in two steps: (i) first we compare the results of SWEET to those given by the Mitsuba renderer [24], this was performed only for the intra-urban scene in day conditions, (ii) then, the foggy images of SWEET are compared to those obtained by the Koschmieder model.

For a more objective comparison, we will use some basic metrics defined in Section 5. We could have made a more objective visual comparison through metrics such as the Visual Differences Predictor (VDP) [41] if we had a reference image (taken by a real camera for the same scene and under the same conditions). This will be added in subsequent work.

## 4. Visual Results

The intra-urban scene (Figure 4a) was simulated in day conditions. The daylight is taken into account in SWEET by setting an airlight radiance value ($1.5\times 10^{7}$ W/m${}^{2}$/sr, which is the radiance of the sun). The camera is a simple model of a perspective pinhole type, with consideration of three wavelengths for RGB channels (700 nm, 550 nm, 450 nm). More complex camera models can be implemented in the future (distortion, spectral responses of RGB filters, Bayer matrix, electronic, etc.).

**Figure 5:**
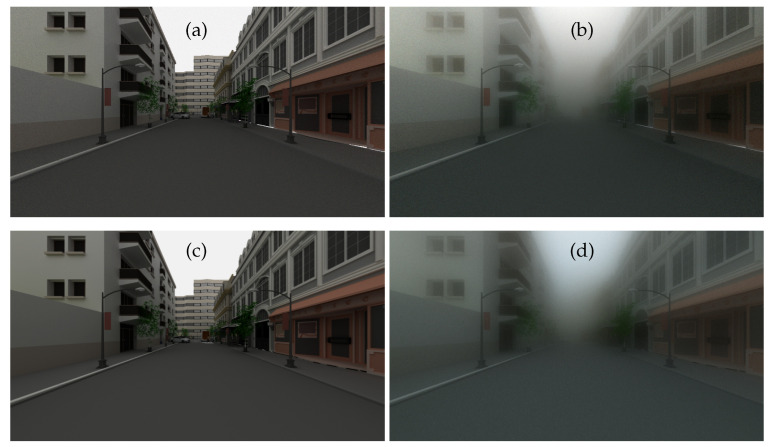
Simulated images for the intra-urban scene in day conditions by the SWEET simulator without fog (**a**) and with fog (MOR = 20 m, (**b**)) and by the Mitsuba renderer without fog (**c**) and with fog (MOR = 20 m, (**d**)).

Figure 5 shows the rendering results obtained by SWEET (a and b) and by Mitsuba (c and d). The same tabbed phase functions as well as the same optical characteristics of the medium (albedo, extinction) are used in the two simulators, corresponding to a visibility of 20 m. These characteristics differ for the colour channels which are calculated separately, then merged to obtain the RGB images of Figure 5.

We note that the renderings of the two simulators are very similar by retaining the same assumptions (without textures, same linear interpolation of colours). Indeed, we did not use the textures in Mitsuba first so that we could compare the results with those of SWEET, which does not integrate the textures, but also because we are more interested in the effects of the fog on the rendering than in the side effects of scene textures. However, we notice the small difference in the colour of the sky in images with fog (Figure 5b,d). This is probably due to slight deviations in the airlight radiance values in Mitsuba for one or more channels. Future investigations will elucidate this point. These results required scene rework and colour re-calibration as Mitsuba is a bit different from SWEET in some aspects. Metrics will be used in the following section to continue the comparison of these images.

**Figure 6:**
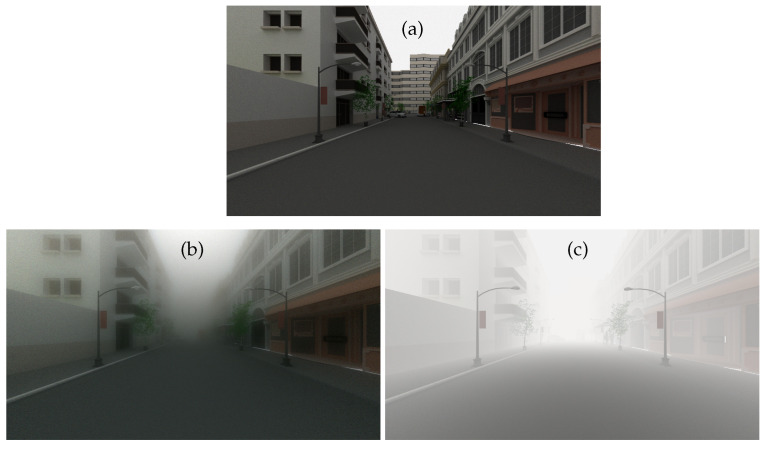
Simulated images for the intra-urban scene with the SWEET simulator without fog (**a**) and with fog (MOR = 20 m, (**b**)) and with the Koschmieder model (**c**) in day conditions.

The images of SWEET without fog are used in the Koschmieder model by considering the same airlight radiance. The images in Figure 6 are obtained with the SWEET simulator without/with fog (a and b) and with the Koschmieder model for the same visibility and the same airlight radiance (c).

At first glance, one can appreciate the realism of the fog produced by the SWEET simulator compared to the Koschmieder model. Indeed, a blur effect can be noticed in the SWEET image, which is not the case for the Koschmieder image.

As we can notice in Figure 6c, Koschmieder’s model brightens the foggy image much more than SWEET (Figure 6b). This can be critical because some objects in the scene are not even visible with the SWEET simulation and are partially visible with the Koschmieder model (e.g., the two pedestrians on the right).

**Figure 7:**
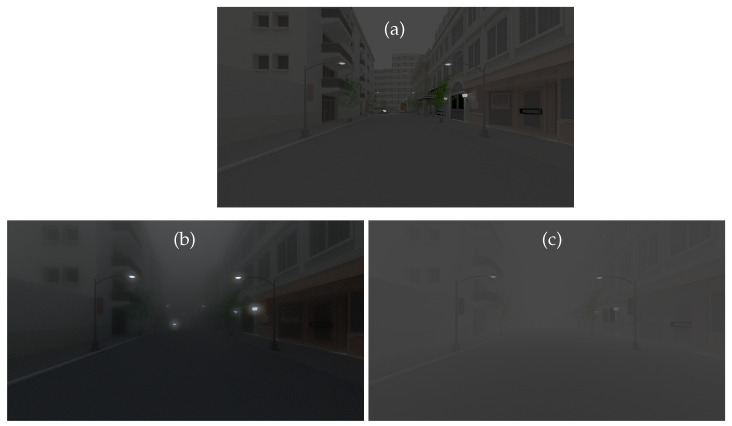
Simulated images for the intra-urban scene with the SWEET simulator without fog (**a**) and with fog (MOR = 20 m, (**b**)) and with the Koschmieder model (**c**) in night conditions.

Now, we compare the models in night conditions, i.e., by considering a very low airlight radiance (5 W/m²/sr) in both models (the full 3D solution of the RTE given by SWEET and the Koschmieder model). The resulting images are given in Figure 7.

Once again, we can notice a nice rendering of the SWEET image (Figure 7b) compared to the one given by Koschmieder (Figure 7c). Indeed, we can observe the halo effects in the former that we do not see in the latter (Koschmieder’s case). This finding was somewhat expected, as it has been identified in the literature that the Koschmieder model is not relevant under night conditions [38]. Contrary to daytime behaviour, the Koschmieder model darkens the rendering at night.

**Figure 8:**
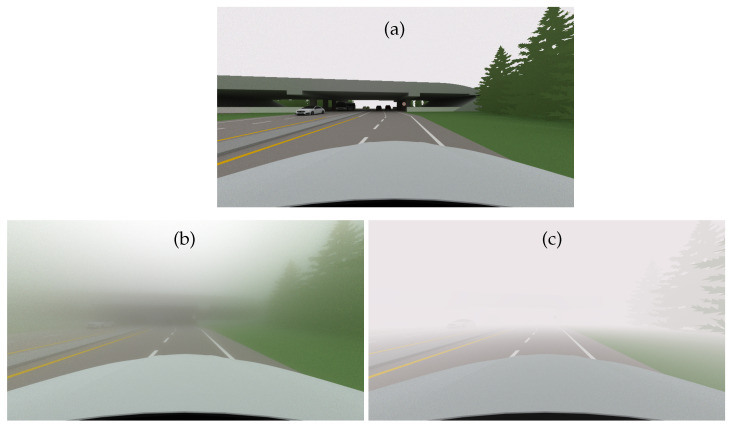
Simulated images for the extra-urban scene with the SWEET simulator without fog (**a**) and with fog (MOR = 20 m, (**b**)) and with the Koschmieder model (**c**) in day conditions.

Figure 8 gives the simulated images by SWEET and the Koschmieder model for the extra-urban scene (Figure 4b). We obtain the same observation as for the intra-urban scene. We can notice that the Koschmieder model brightens too much under the viaduct (Figure 8c), while this is not the case for the image simulated by SWEET (Figure 8b). Moreover, the latter allows more objects to be seen (i.e., the car in front, trees, the viaduct) which are totally invisible with the Koschmieder model.

**Figure 9:**
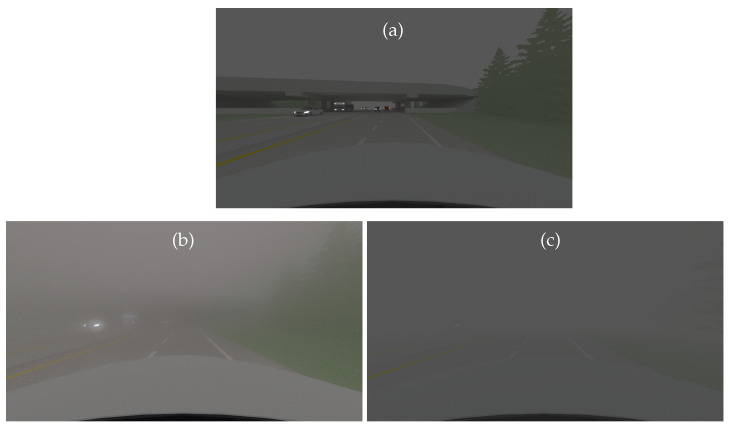
Simulated images for the extra-urban scene with the SWEET simulator without fog (**a**) and with fog (MOR = 20 m, (**b**)) and with the Koschmieder model (**c**) in night conditions.

Figure 9 gives the simulations of the extra-urban scene in night conditions.

The halo effects are visible in the SWEET image (Figure 9b) and not in the Koschmieder one (Figure 9c). In fact, as mentioned above, the Koschmieder model darkens too much at night.

We have visually seen that the images obtained differ markedly between the Koschmieder and SWEET models. From a subjective point of view, we can say that the SWEET model brings a lot of improvement, thanks to its integration of 3D volumetry. We now propose to verify the impact of the blurring on a realistic image. Then, we propose to set up metrics to confirm what we perceive visually thanks to a discussion of quantitative metrics on real and simulated images in the next section.

**Figure 10:**
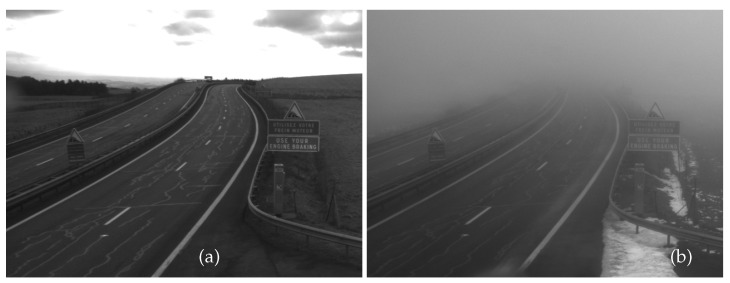
Real camera images taken from a section of the French A75 highway (at the Col de la Fageole point) without fog (**a**) and with fog (MOR = 156 m, (**b**)), both images are in grayscale.

The images in Figure 10 show a section of the French A75 highway, at the Col de la Fageole point, without fog (a) and with fog (b) (see [42] for a detailed description of the database). The image with fog (Figure 10b) can be compared to the simulated images (Figure 6b and Figure 8b). Although the latter are not made in the same scene, it still allows us to observe a similarity in the visual aspect of the fog in all these images (i.e., blurry effects). This confirms the realism of the rendering performed by SWEET. In perspective, we will then propose simulations on this specific use case by reproducing the same scene as that of the Col de la Fageole. Meanwhile, we propose to compare the general behaviour obtained on real and metrically simulated images in the next section.

## 5. Quantitative Results with Specific Metrics

In this section, we push the interpretation of the images even further, and we then propose analyses by two metrics:

- intensity analysis on lines of pixels;
- contrast analysis.

**Figure 11:**
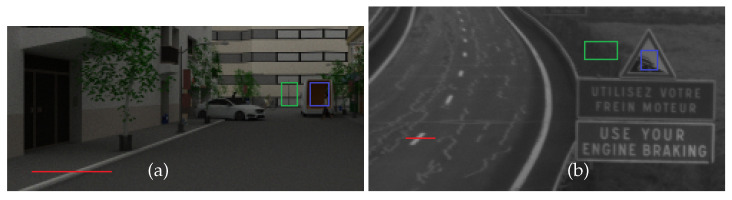
Pixel lines (in red) and contrast polygons (in blue/green) for the urban simulated scene (**a**) and the real one from a section of the French A75 highway (at the Col de la Fageole point) (**b**).

First, we present in the images of Figure 11, the specific regions where we will calculate the metrics for both real and simulated images. The pixel lines in red are used in order to verify the blurring effect produced by the fog on areas of sudden change of intensity (contours, ground markings, edge of building, etc.). The polygons shown in blue/green are used to compute the contrast according to the WMO definition [38]:

(9) $$C=\frac{L_{b}-L_{h}}{L_{h}},$$

where C is the contrast, $L_{b}$ is the radiance of the object (blue polygons) and $L_{h}$ the radiance of the surrounding object area (green polygons).

**Figure 12:**
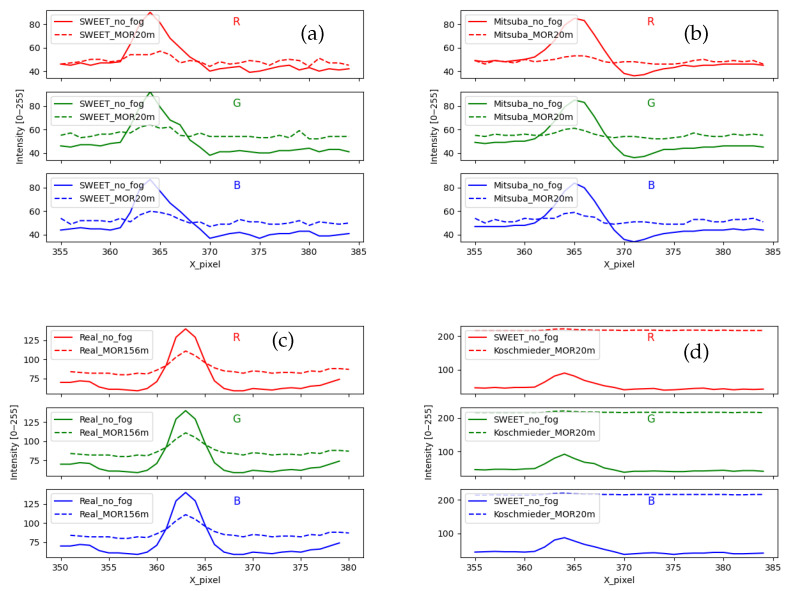
Comparing the intensity of a pixel line for images coming from SWEET (**a**), Mitsuba (**b**), real camera images (**c**) and Koschmieder (**d**).

We start by analysing the evolutions of the pixel lines. The curves in Figure 12 are obtained for SWEET images (a), Mitsuba images (b), real images (c) and Koschmieder images (d). The peak in all the curves without fog (solid lines) represents the studied object, i.e., marking on the ground in the case of real camera images (Figure 11b) and the edge of the sidewalk in the case of the intra-urban road simulated scene (Figure 11a).

We can notice the similar behaviour of SWEET and Mitsuba through Figure 12a,b. However, Sweet images contain a little more noise, which can be eliminated in the future using denoising and sampling techniques. Moreover, we can notice through the solid curves in Figure 12b that the peak is smoother than in (a), which looks more like the curves of the real images (c).

The presence of fog in the scene simulated by SWEET, Mitsuba and in the real road scene attenuates the peak representing the object (ground markings, edge of sidewalk) and slightly lightens the scene around as can be seen through the curves in the broken line of Figure 12a–c. However, in the case of the Koschmieder model, the behaviour is completely different, and this again confirms that this model excessively brightens the whole image as can be seen in Figure 12d.

**Figure 13:**
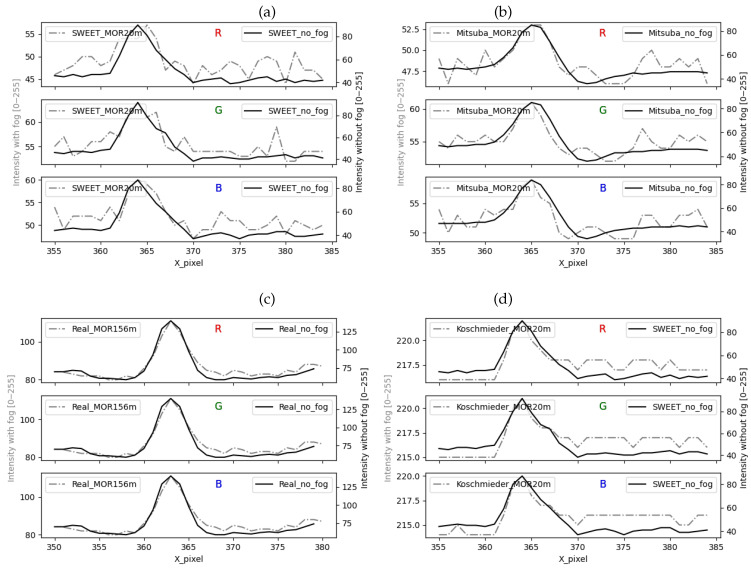
Comparing the intensity of a pixel line for images coming from SWEET (**a**), Mitsuba (**b**), real camera images (**c**) and Koschmieder (**d**), rescaled figures.

The curves with/without fog in Figure 12 are rescaled (separate y-axis for curves in solid/broken line), giving rise to Figure 13. We can immediately see that the rise towards the peak of the discontinuous curves is not identical. Indeed, the image with fog simulated by SWEET and Mitsuba (discontinuous curve in Figure 13a,b) presents a gradual rise like the real case (discontinuous curve in Figure 13c). On the other hand, a very sharp slope is observed in the case of Koschmieder (discontinuous curve in Figure 13d). This confirms the presence of the blur effect in the images simulated by SWEET, which is a realistic effect, which is not the case for Koschmieder.

**Figure 14:**
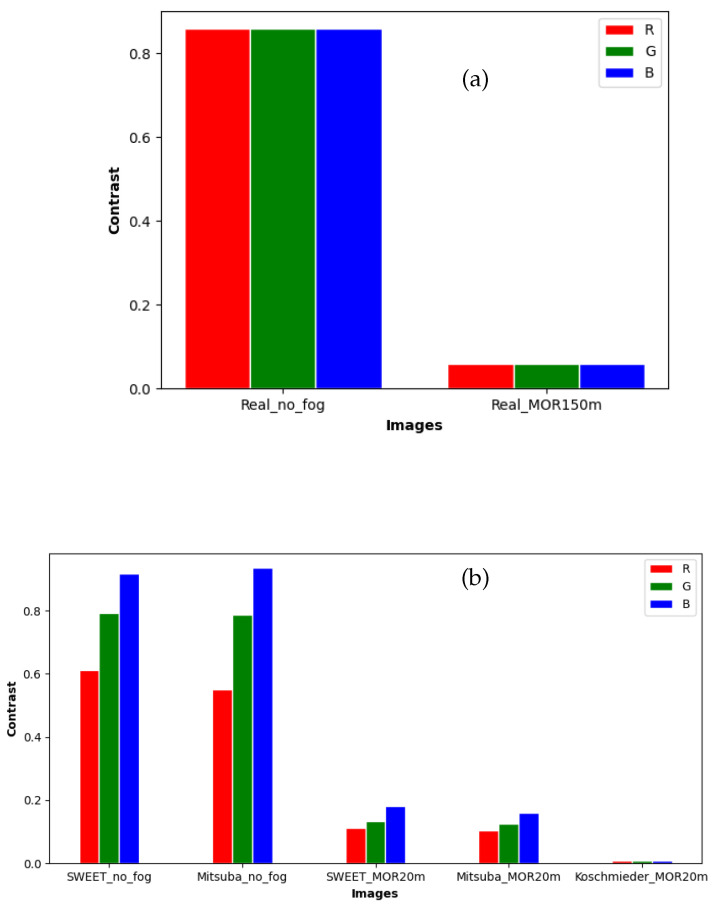
Comparing the contrast of objects for images from real images (**a**) to that of simulated images (SWEET, Mitsuba and Koschmieder) (**b**).

Contrast levels of the image simulated by SWEET and Mitsuba are reduced similarly for the three colour channels (red, green and blue) when the fog is applied, whereas in the case of Koschmieder, the contrast levels are almost zero as shown in Figure 14b. By comparison with the results of the real images (Figure 14a), this shows once again that the SWEET simulation of foggy images is more relevant.

The contrast levels for the real scene (Figure 14a) are identical for the three channels (R, G and B). This is because the images of the real scene (Figure 10) are in grayscale.

**Figure 15:**
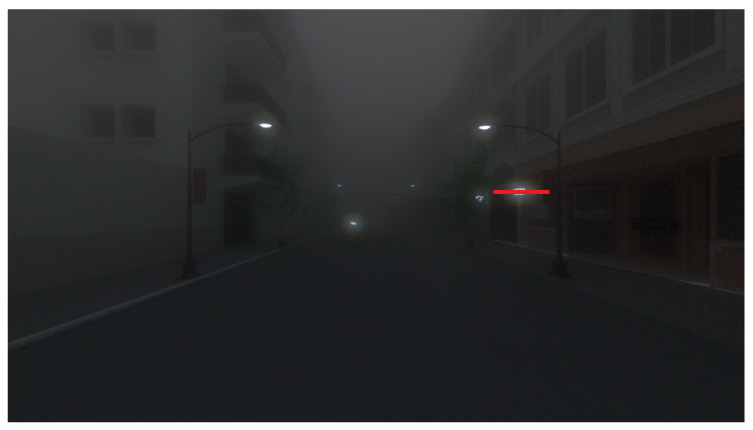
Pixel line (in red) for the urban simulated scene at night.

We now propose to verify the halo phenomenon by using the line pixels comparison on the night images (only SWEET is used). Figure 15 shows the simulated urban scene by SWEET. In the following, we propose to analyse the halo effects more closely through the pixel line in red.

**Figure 16:**
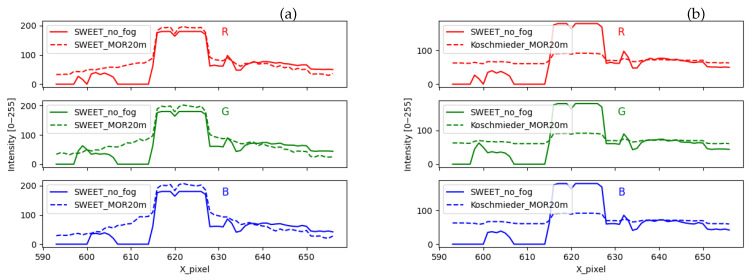
Comparing the intensity of a pixel line for the urban scene simulated by the SWEET simulator (**a**) and Koschmieder (**b**).

The peak of the curves in Figure 16 located in the pixel interval [617–628] corresponds to the white light of the lamp. The intensity of that white light is mostly preserved in the foggy image simulated by SWEET (broken line curves in Figure 16a). However, that peak is almost entirely attenuated by the Koschmieder model (broken line curves in Figure 16b). Around the lamp, the curve with fog simulated by SWEET (broken line curves in Figure 16a) has a gradient slope representing the effect of the halo. This is not the case with the Koschmieder model for which the curve gives almost a constant (broken line curves in Figure 16b).

**Figure 17:**
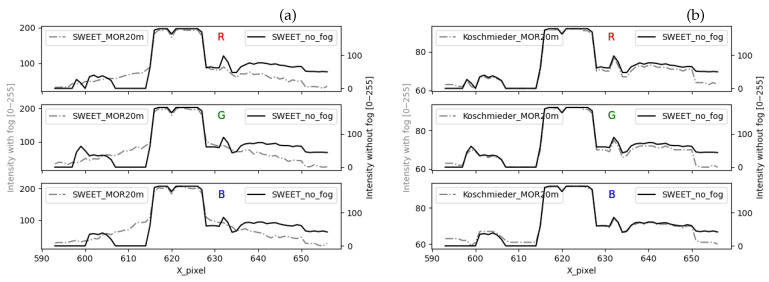
Comparing the intensity of a pixel line for the urban scene simulated by the SWEET simulator (**a**) and Koschmieder (**b**), rescaled figures.

The shape of the discontinuous curve in Figure 17b is the same as the one without fog. This confirms that the Koschmieder model darkens the whole image. On the other hand, we can notice the gradient slope given by the SWEET simulator (discontinuous curve in Figure 17a).

## 6. Conclusions and Perspectives

For the evaluation and design of optical sensors for automated vehicles, digital simulation is a tool that can be used to approach reality in numerous configurations and with different degrees of realism. In this context, the SWEET simulation tool developed by Cerema meets the needs of research into the simulation of road scenes under degraded weather conditions, including fog. The choice of Monte Carlo methods (ray-tracing) in this simulator is justified by the accuracy of the results and the expected realism of the simulated scenes. Other methods based on simplified models of electromagnetic propagation through fog can be used as they can reach real-time execution.

In this paper, SWEET was first compared to the Mitsuba simulator. This comparison with a visual approach, then with some basic metrics, showed that the two simulators gave very close renderings. Improvements can be made to SWEET to reduce noise and smoothen curves as noted through Figure 12a–c. After that, SWEET and Mitsuba were both compared to the simplified Koschmieder model, which is widely used in the simulation of fog effects in images. The visual comparison of the images obtained by the two models (i.e., SWEET vs. Koschmieder) highlighted the realism of SWEET, which is able to reproduce real effects observable in road scene images (i.e., blurring effect, halo effect, etc.) as was seen through Figure 6b,c, Figure 7b,c, Figure 8b,c and Figure 9b,c. These visual observations were then quantified using metrics on pixel lines and comparison of contrast levels of several objects in the scenes. The results obtained in this work show a good match of the metrics between real fog images and images from the SWEET simulator as can be noted in Figure 12a compared to Figure 12c. The Koschmieder model shows deviations from the real images as can be seen in Figure 12d.

Future work will consist of continuing the comparison between SWEET, Mitsuba and the Koschmieder model on a wider variety of images, for other levels of fog and for other metrics of interest that could be built on higher level indicators, such as VDP or obstacle detection algorithms, for example. The Koschmieder model could be extended by adding the consideration of halo effects, blurring, etc. as has been performed in [26] in order to be able to use it in support of other simulators while guaranteeing satisfactory realism. Moreover, it would be interesting to perform more applications using common datasets. It will also be useful to compare the physical radiance outputs of the SWEET simulator with experimental measurements acquired with a spectroradiometer or a hyperspectral camera using the PAVIN platform or real-world sites instrumented by Cerema and its partners. Furthermore, ongoing work would complete future versions of SWEET with textures and BSDFs in order to get as close as possible to physical reality. Another perspective will be to build a database of images generated by SWEET in order to train machine learning algorithms able to deliver harsh weather noise models that can be run in real time. The work may also be extended to other wavelengths for cameras operating in SWIR and LWIR ranges or to active technologies such as LiDAR and RADAR.

## Data Availability

Not applicable.

## Abbreviations

The following abbreviations are used in this manuscript:

SWEET	Simulating WEather for intElligent Transportation systems
PAVIN	Plateforme d’Auvergne Véhicules Intelligents
LiDAR	Light Detection And Ranging
RADAR	RAdio Detection And Ranging
RTE	Radiative Transfer Equation
DSD	Droplet Size Distribution
MOR	Meteorological Optical Range
SWIR	Short-Wave InfraRed
LWIR	Long-Wave InfraRed
